# An “In-Depth” Description of the Small Non-coding RNA Population of *Schistosoma japonicum* Schistosomulum

**DOI:** 10.1371/journal.pntd.0000596

**Published:** 2010-02-09

**Authors:** Zhangxun Wang, Xiangyang Xue, Jun Sun, Rong Luo, Xindong Xu, Yanyan Jiang, Qingfeng Zhang, Weiqing Pan

**Affiliations:** 1 Institute for Infectious Diseases and Vaccine Development, Tongji University School of Medicine, Shanghai, China; 2 Department of Pathogenic Biology, Second Military Medical University, Shanghai, China; University of Pittsburgh, United States of America

## Abstract

Parasitic flatworms of the genus *Schistosoma* are the causative agents of schistosomiasis, which afflicts more than 200 million people yearly in tropical regions of South America, Asia and Africa. A promising approach to the control of this and many other diseases involves the application of our understanding of small non-coding RNA function to the design of safe and effective means of treatment. In a previous study, we identified five conserved miRNAs from the adult stage of *Schistosoma japonicum*. Here, we applied Illumina Solexa high-throughput sequencing methods (deep sequencing) to investigate the small RNAs expressed in *S. japonicum* schistosomulum (3 weeks post-infection). This has allowed us to examine over four million sequence reads including both frequently and infrequently represented members of the RNA population. Thus we have identified 20 conserved miRNA families that have orthologs in well-studied model organisms and 16 miRNA that appear to be specific to *Schistosoma*. We have also observed minor amounts of heterogeneity in both 3′ and 5′ terminal positions of some miRNA as well as RNA fragments resulting from the processing of miRNA precursor. An investigation of the genomic arrangement of the 36 identified miRNA revealed that seven were tightly linked in two clusters. We also identified members of the small RNA population whose structure indicates that they are part of an endogenously derived RNA silencing pathway, as evidenced by their extensive complementarities with retrotransposon and retrovirus-related Pol polyprotein from transposon.

## Author Summary

Schistosomiasis is one of the most prevalent and serious parasitic diseases in tropical and subtropical regions. Schistosomes are pathogens that have a unique repertoire of genes expressed at different life cycle stages. Small regulatory RNAs play an important role in the control of gene expression at the post-transcriptional level. In a previous study, we identified five conserved miRNAs in adult *S. japonicum* worms through traditional sequencing methods. In this work, we discovered more microRNAs, including 16 new schistosome-specific miRNA families in addition to 20 conserved miRNA families. We identified two miRNA gene clusters using the data obtained from high-throughput sequencing. We demonstrated, for the first time, the presence of endogenous small interfering RNAs in schistosomes. Identification of these small regulatory RNAs should provide a foundation for future studies aimed at understanding functions of small RNAs and ultimately of small RNA regulatory networks in this important pathogen.

## Introduction

Small RNAs constitute a family of regulatory non-coding RNAs 19–28 nt in length. These serve to modulate the translation of messenger RNA (mRNA), establish chromosomal architecture, and provide defense against viruses and mobile genetic elements (transposons) [1]–[3]. Three categories of regulatory non-coding RNA have been established based on features involving their origin, structure and biological role: small interfering RNAs (siRNAs), microRNAs (miRNAs), and piwi-interacting RNAs (piRNAs) [4].

MiRNAs are generated from precursor transcripts by the action of two RNase III-type proteins, Drosha and Dicer. Drosha cleaves primary transcripts (pri-miRNA) yielding an approximately 60–80 nucleotide (nt) stem loop intermediate known as the precursor miRNA (pre-miRNA) [5],[6]. The pre-miRNA is further cleaved by Dicer to release the miRNA/miRNA* duplex [6],[7]. One strand of the RNA duplex, the miRNA, is stably incorporated into the RNA-induced silencing complex (RISC) while the other strand, the miRNA*, is degraded. The RISC, loaded with miRNA, targets mRNAs and functions as a post transcriptional regulator [8]. In animals, the incorporated mature miRNA guides RISC to repress the expression of target mRNA through partial complementarities with the 3′ -UTR of the target mRNA. Most miRNAs contain a 7 nucleotide region (positions 2–8 of the miRNA) known as the miRNA “seed sequence” [9] that is complementary to its target mRNA(s). Although it is now evident that animal miRNAs can also direct the degradation of their target mRNAs [9], few animal miRNAs seem to be sufficiently complementary to mRNAs to initiate what is referred to as the Slicer mechanism. This “silencing effect” appears to be related to the extent of complementarity [9] between the miRNA and its target.

Endogenous small interfering RNAs (Endo-siRNAs) are generated from long double stranded RNAs (dsRNA). These dsRNA can be the product of bi-directional transcription of genomically encoded sequence producing both sense and antisense strands. Endo-siRNAs could also originate from protein-coding genes whose transcripts can pair with transcripts of related pseudogenes [4].Long dsRNA is a substrate for Dicer, but not for Drosha [10]. Dicer must make two successive pairs of cuts to yield a siRNA duplex. The siRNA-specific RISC assembly machinery selectively loads the guide strand into RISC and the passenger strand is degraded. The mature endo-siRNA are nearly always 21 nts in length, have modified 3′ termini, and unlike miRNAs and piRNAs are not biased towards beginning with uracil [11]. Their complementarity with the target RNA is associated with cleavage and silencing of the target RNA [12]. The first mammalian endo-siRNAs to be reported was shown to be complementary to the long interspersed nuclear element (L1) retrotransposon [4].

The longest of the three classes, piRNAs (24–31nt in length) have been described in Drosophila and mammals, are associated with Piwi subfamily proteins, and are highly abundant in germ cells [4],[11],[12]. The derivation of piRNAs from precursors remains poorly understood but appears to involve a single-stranded RNA and is not dependent on Dicer [12],[13]. At least some piRNAs are involved in transposon silencing through heterochromatin formation or RNA destabilization [12].

The genus *Schistosoma* includes three species (*Schistosoma japonicum*, *Schistosoma mansoni*, and *Schistosoma haematobium*), which are the major causes of human schistosomiasis, one of the most prevalent and serious parasitic diseases in tropical and subtropical regions. The complex life cycle of schistosomes involves multiple developmental stages, including egg, miracidium, cercaria, schistosomulum and adult worm. The newly generated information on the *S. japonicum* and *S. mansoni* genomes will serve as a foundation for the identification of small regulatory RNAs in the genus *Schistosoma* [14],[15]. Previously we identified 5 miRNAs in adult *S. japonicum* worms by sequencing cDNA libraries made from small RNA [16]. Recent advances in high-throughput sequencing technology have allowed for a more complete assessment of the global small RNA population. These studies permit not only qualitative and quantitative studies of abundant small regulatory RNAs, but also have allowed us to identify small regulatory RNAs expressed at much lower levels [17],[18]. Here, we describe the small RNA population of the schistosomulum stage of *S. japonicum*.

## Materials and Methods

### Parasite culturing

Parasite culturing was performed as described previously [16]. Briefly, hepatic schistosomula were recovered by perfusion from BALB/c mice that had been infected 3 weeks earlier with 100 cercariae. All procedures performed on animals within this study were conducted in accordance with and by approval of the Internal Review Board of Tongji University School of Medicine. Cercariae of *S. japonicum* were shed from snails (*Oncomelania hupensis*), provided by the National Institute of Parasitic Disease, Chinese Center for Disease Control and Prevention. After collection, all freshly isolated samples were washed three times with 1× Phosphate buffered saline (PBS) pH 7.4 and were immediately used for extraction of total RNA or stored in liquid nitrogen.

### Construction of Small RNA libraries and sequencing

Total RNA was extracted from schistosomula using Trizol (Invitrogen). A 20 µg aliquot was enriched for small RNA using the PEG8000 precipitation method [19]. The small RNAs between 18–30 nucleotide (nt) were isolated by polyacrylamide gel electrophoresis (PAGE ). This resulting fraction of RNA was ligated to Illumina's proprietary 5′ and 3′ adaptors and the products were amplified by RT-PCR. The purified PCR products were used for clustering and sequencing by an Illumina Genome Analyzer at the Beijing Genomics Institute, Shenzhen.

### Sequence analysis

All unique sequences along with their associated read counts were determined from the raw data. The unique sequences were mapped to the *S. japonicum* genome (http://www.chgc.sh.cn/japonicum/Resources.html) and the *S. mansoni* genome (http://www.sanger.ac.uk/Projects/S_mansoni) using WU-BLAST software [20]. To remove unique sequences originating from rRNA, tRNA, snRNA(small nuclear RNA), and snoRNA(small nucleolar RNA), we used the sequences of noncoding RNAs collected in Rfam 9.0 [21] and the NCBI GenBank data (http://www.ncbi.nlm.nih.gov/).

The identification of *S. japonicum* miRNAs was carried out using previously established criteria [16],[18]. Briefly, we identified all small RNA sequences with the potential to form hairpin-like structures using RNAfold [22],[23]. We eliminated all predicted hairpin-like structures having a minimum free energy more than or equal to −20 kcal/mol as well as those with bulges bigger than 4 nucleotides and multiple loops. The candidates with a corresponding miRNA* form were identified directly as miRNAs.

Candidate *S. japonicum* endo-siRNAs were identified using criteria similar to those used for *Caenorhabditis elegans* endo-siRNAs [24],[25]. Briefly, annotated *S.japonicum* sequence data sets were downloaded from the LSBI *S.japonicum* Web site (http://www.chgc.sh.cn/japonicum/Resources.html). Small RNA sequences that overlapped predicted intron or protein-coding exons were obtained by an integrated analysis with the data of Genome scaffolds and Predicted coding genes. Those small RNAs perfectly matching the exon-antisense strand were considered as candidate endo-siRNAs.

### Northern blot analysis

Northern blot analysis was carried out as described previously [16],[26]. DNA oligonucleotides complementary to small RNA sequences were end-labeled with biotin at 5′ Termini (Invitrogen, Shanghai) and used as probe (Table S7). Blots were washed four times (two times with 2× SSC(Sodium chloride and Sodium Citrate solution) +1% SDS(Sodium Dodecyl Sulfate) at room temperature and two times with 0.5× SSC +0.5% SDS at 48°C). Hybridization was detected using a North2South Kit (Pierce) following the manufacturer's instructions. Signals were visualized using a Kodak image station 2000. All blots shown are representative of at least three independent experiments.

### Quantitative RT-PCR of miRNAs expression analysis

A stem-loop qRT-PCR method was used to quantitate miRNA expression [16]. A stem-loop RT primer was used to reverse-transcribe mature miRNAs to cDNAs. The 20 µl reverse-transcription reactions contained 1µg of total RNA, 50 nM of each individual stem-loop RT primer, 0.5 µM dNTP (Takara), 5 U M-MLV reverse transcriptase (Takara), and 2 U RNase inhibitor. The temperature program was 30 min at 16°C, 30 min at 42°C, 15 min at 70°C and then held at 4°C.

We then used real-time PCR to monitor and quantify miRNAs using qRT-PCR [16],[27]. Real-time quantification was performed using an Applied Biosystems 7300 Sequence Detection system. The 20 µl PCR reaction included 2 µl of RT product (1∶1 dilution), 1× SYBR Premix Ex Taq II (Takara), 0.5 µM specific forward primer, 0.5 µM common reverse primer. The reactions were incubated in a 96-well plate at 95°C for 10 sec, followed by 40 cycles of 95°C for 5 sec and 60°C for 31 sec.

For relative quantification of 3 miRNAs originating from a single genomic cluster, the ΔΔCt method was employed [28], using U6 RNA as the internal control for each sample. All reactions were run in triplicate. All primers used are listed in Table S8.

### Analysis of RNA 3′ termini

Periodate oxidation and β-elimination of RNAs were performed as described [29],[30]. The RNAs were precipitated in ethanol and analyzed by northern blot. Biotin-labeled probes were used for the analysis—endo-siRNA-3: 5′- TGCCTCTGCCTCCCGAGTGC-3′, miR-307:5′-CTCAATCAAGTAGGTTGTGA-3′.

## Results

### Overview of *S. japonicum* schistosomulum small RNA library sequencing

RNA was isolated from *S. japonicum* schistosomulum (3 weeks post-infection) and fractionated on the basis of size. We prepared a library for sequencing (as described in the Methods section of this manuscript) from the fraction containing small RNA 18–30 nucleotides in length. High-throughput sequencing, using Illumina/Solexa technology, yielded 4,344,045 quality reads that could be mapped to the *S. japonicum* genome(Table S1). Analysis of this data indicated that the library represented a diverse population of small RNAs whose members differed in sequence, copy number and extent of sequence homology with small RNAs from other eukaryotic organisms. Categorization of all sequence indicated that 30% have structural features characteristic of miRNA, 35% matched annotated noncoding RNA genes such as rRNAs, tRNAs, snRNAs, and snoRNAs (Table 1) while the remaining 35% could not be identified.

**Table 1:** The summary distribution of small RNAs.

Small RNA Categories	Unique sRNAs*	Percentage of Unique sRNA Reads (%)	Total sRNAs (Reads)	Percentage of Total sRNA Reads (%)
**miRNAs**				
Known miRNA	5	0.0016	400,107	9.21
Conserved miRNA	20	0.0064	928,336	21.37
Schistosome-specific miRNA	16	0.0051	6,067	0.14
Candidate miRNA	21	0.0067	2,001	0.05
endo-siRNAs	4,858	1.5630	59,669	1.3736
**Nonprotein-coding RNAs**				
rRNA	46,374	14.9201	1,473,101	33.91
tRNA	3,014	0.9697	56,955	1.31
snRNA	1,082	0.3481	4,945	0.11
snoRNA	43	0.0138	72	0.00
Other small RNAs**	255,383	82.1653	1,412,792	32.5225
**Total**	310,816	100	4,344,045	100

*indicates the number of unique sRNAs.

**indicates sequences that do not match any known miRNAs, endo-siRNAs or nonprotein-coding RNAs.

Over one million different primary sequences were detected with the copy number of the individual sequence ranging from 584,105 to 1. Variation in the number of times that each sequence is detected reflects its relative copy number in the library and, under ideal conditions, the relative abundance of these small RNAs in the schistosomulum.

Over 65,000 of the unique sequence types gathered from *S. japonicum* were also homologous with regions of the *S. mansoni* genome indicating the presence of an important subdivision: 1) “conserved RNAs” that share significant homology with the miRNAs of other related species and 2) “species-specific RNAs” whose primary sequence has not been reported in other species but which have the structural features that are characteristic of miRNA.

### The abundant forms of miRNA in *S. japonicum*

Five of the *S. japonicum* miRNAs (sja-let-7, sja-miR-71, sja-bantam, sja-miR-125 and sja-miR-new1) reported here have been previously identified and characterized [16]. These 5 miRNA sequences accounted for 30% of the sequence reads determined to be miRNA. Further, a single and newly identified miRNA (miR-1a) alone accounted for 43% of total sequence having the characteristics of miRNAs. In total, we found that the 6 most abundant miRNAs accounted for approximately 73% of the total read counts of all putative miRNA sequences in our library.

**Figure 1:**
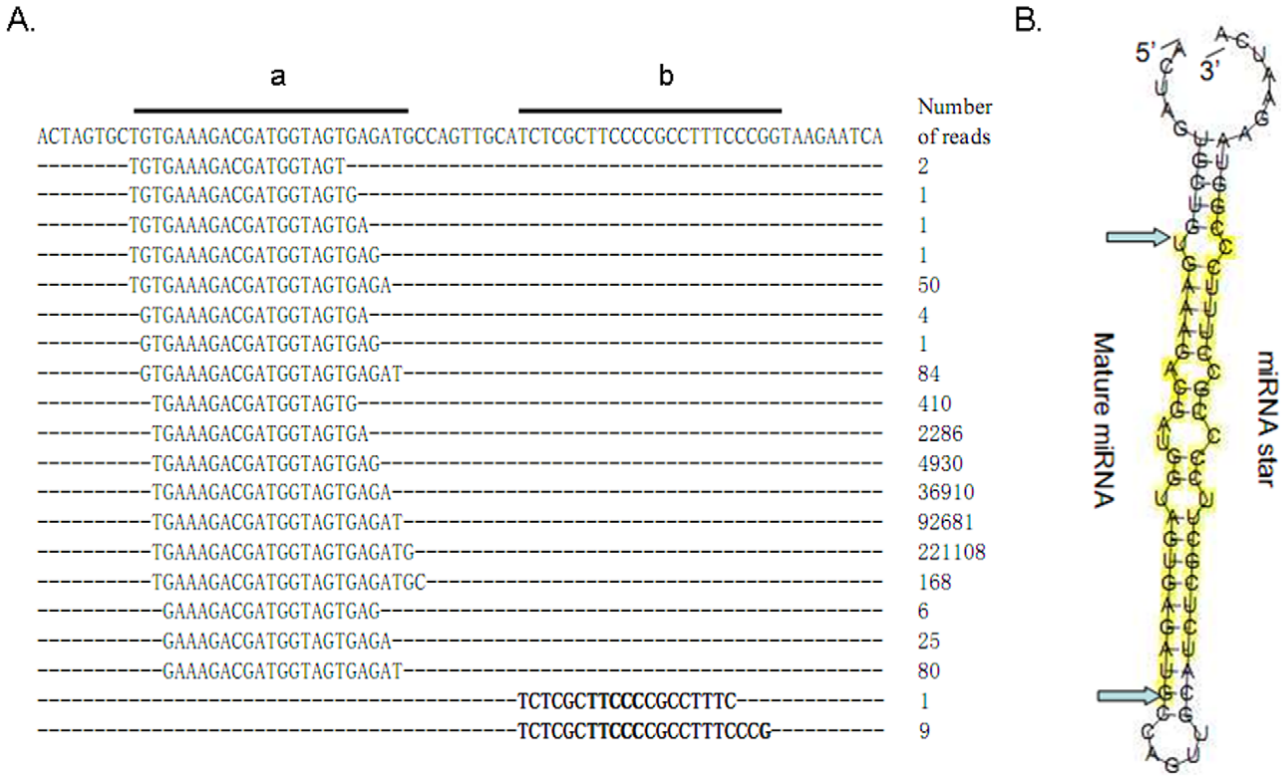
Prediction of miRNA candidates on the basis of primary sequence and the secondary structure of the putative pre-miRNA. (**A**) Prediction of miRNA candidates on the basis of primary sequence. A candidate pre-miRNA gene sequence is shown with the miRNA (a) and miRNA*(b) sequences noted. An alignment of unique sequences related to the pre-miRNA gene is shown below along with the number of times it was detected (read count) by high throughput sequencing. Sequences relating to miRNA* are bolded. This example is based upon a 74 nucleotide sequence which includes **sja-miR-71a** (location: CAC|CCON0000000053.1|:242744:242817). (**B**) The secondary structure of the putative pre-miRNA. The sequence encoding miRNA was based on prevalence in the library. Secondary structure was predicted based upon the identification of the miRNA and predicted on the theoretical folding criteria established in other systems (reference 18) using the RNA fold algorithm(reference 22,23). Detection of sequences in our RNA library corresponding to the predicted miRNA* serves to support the predicted structure.

Sequencing of small RNA libraries by traditional cloning methods often reveals the highly abundant miRNAs. Using high-throughput deep sequencing we also detect sequences that are related to the abundant forms but with very low copy numbers. These can also yield important biological information about the related abundant forms. For example, we observed heterogeneity at the 5′ and 3′ ends of abundant miRNAs, a phenomenon that has previously been noted [17],[31]. We document the type of heterogeneity because of its potential biological significance as will be discussed below. We refer to RNAs resulting from variation from their “reference” miRNA sequences at the termini as isomiRs (Figure 1).

### Approaches to the identification of less abundant miRNAs in *S. japonicum*

#### (1) Interspecies sequence conservation

To identify candidate miRNAs in *S. japonicum* we compared our sequences with miRNAs of other organisms by doing a similarity search (BLASTN with an E-value cutoff of 10) using the Sanger miRNA Registry database [32] as a resource. These criteria have been used in several recent miRNA studies [33],[34]. A total of 20 miRNAs belonging to 18 “conserved miRNA” families were identified (Table 2 and Table S3). The majority of novel miRNAs appeared to have significant sequence homology within *S. mediterranea*, the genome most closely related to the genus *Schistosoma* (Table 2). This observation is consistent with the proposal that *S. japonicum* and *S. mediterranea* share common features of the phylum Platyhelminths [35].

**Table 2:** Conserved *S. japonicum* miRNA families homologous to known miRNAs from other animal species.

Family	Name	miRNA Sequence(5′-3′)*	Length	Reads	Conserved in Other Animal Species		
					*S. mediterranea*	*C. elegans*	*D. melanogaster*
miR-1	sja-miR-1a	UGGAAUGUUGUGAAGUAUGUGC	22	584105	miR-1b/c		
	sja-miR-1b	UGGAAUGUGGCGAAGUAUGGUC	22	552	miR-1b		miR-1
miR-2**	sja-miR-2a	UCACAGCCAGUAUUGAUGAACG	22	13431	miR-2b	miR-2	miR-2a/c
	sja-miR-2b	UAUCACAGCCCUGCUUGGGACACA	24	57	miR-2a		
miR-7	sja-miR-7	UGGAAGACUGGUGAUAUGUUGUU	23	80869	miR-7b/c		miR-7
miR-8**	sja-miR-8	UAAUACUGUUAGGUAAAGAUGCC	23	143	miR-8	miR-236	miR-8
miR-10**	sja-miR-10	AACCCUGUAGACCCGAGUUUGG	22	1002	miR-10		miR-10
miR-31	sja-miR-31	UGGCAAGAUUACGGCGAAGCUGA	23	31			miR-31a
miR-36**	sja-miR-36	CCACCGGGUAGACAUUCAUUCGC	23	1478	miR-36		
miR-61	sja-miR-61	UGACUAGAAAGUGCACUCACUU	22	1712	miR-61		
miR-71**	sja-miR-71b	UGAAAGACUUGAGUAGUGAGACG	23	196399	miR-71c		
miR-87	sja-miR-87	GUGAGCAAAGUUUCAGGUGUGU	22	50	miR-87a	miR-87	miR-87
miR-124**	sja-miR-124	UAAGGCACGCGGUGAAUGUCA	21	31185	miR-124a/c	miR-124	miR-124
miR-133	sja-miR-133	UUGGUCCCUAUCAACCAGCUGU	22	2	miR-133		miR-133
miR-190	sja-miR-190	UGAUAUGUAUGGGUUACUUGGUG	23	6786	miR-190a/b		
miR-219	sja-miR-219	UGAUUGUCCAUUCGCAUUUCU	21	5448	miR-219		miR-219
miR-277	sja-miR-277	UAAAUGCAUUUUCUGGCCCG	20	3272	miR-277b/d		
miR-281	sja-miR-281	UGUCAUGGAGUUGCUCUCUAU	21	1800		miR-46/47	miR-281
miR-307	sja-miR-307	UCACAACCUACUUGAUUGAG	20	4			miR-307
miR-310	sja-miR-310	AUAUUGCAAAUUCCCGGCCUUU	22	10			miR-310

*Corresponding secondary structures are listed in Table S3.

**indicate those *S. japonicum* miRNAs that are conserved in *S. mansoni*.

#### (2) Conservation of secondary structure

A number of *S. japonicum* miRNAs were identified using commonly accepted criteria that is based on predicted secondary structure [24],[31],[36]. Hence many of the sequences could be eliminated from consideration as candidate miRNAs because their primary sequence would not allow formation of the characteristic pre-miRNA stem-loop structure. For the remaining RNA sequences, strong supporting evidence for the designation of miRNA is often provided by deep sequencing technology and the massive sampling power it provides. For example, mature miRNAs are formed from a primary transcript as the result of a predictable series of steps. With sufficient sampling both mature forms and predicted intermediates are found in the collected sequence reads. In the case of miRNA the stem-loop precursor transcript is cleaved by the nuclease, Dicer, leaving a small dsRNA and the single stranded RNA that constituted the loop. Subsequent activity results in the selection of one strand of the dsRNA (the miRNA) to be part of the silencing complex while the other strand (miRNA*) and the loop structure are degraded. However as a result of extensive sampling, the miRNA* for 16 novel sequences were found indicating Dicer activity associated with the candidate miRNA. This provides compelling, albeit indirect, evidence for Dicer-like processing from an RNA hairpin precursor [24],[31],[36] and thus lending support to the identification of the candidate sequences as a miRNAs. In addition to the identification of the miRNA* strand, we were able to identify the loop sequences of a small number of pre-miRNAs hence accounting for all products of Dicer cleavage(let-7, bantam in Table S2). Such information is of value in determining the nature of enzymatic activity in the cell and will be critical for the design of transfection vector for the introduction of miRNAs into live cells.

Sixteen sequences met all these criteria and were designated novel miRNAs (Table 3). The predicted precursor structures as well as the sequences of miRNA* forms corresponding to novel miRNAs are provided in Table S4. Among 16 novel miRNAs, 7 sequences begin with a 5′ uridine, which is a characteristic feature of miRNAs. In addition, we identified another 21 candidate miRNAs which having the appropriate length (20–24 nt) and meeting the hairpin characteristics criteria did not display the characteristics of miRNA biogenesis (Table S5), and thus will require additional validation.

**Table 3:** Schistosome-specific miRNAs identified by deep sequencing.

Name*	miRNA Sequence (5′-3′)*	Length	reads
sja-miR-novel-01	UAUUAUGCAACGUUUCACUCU	21	4744
sja-miR-novel-02	UAUUGCACUUACCUUCGCCUUG	22	809
sja-miR-novel-03	ACCCUUGUUCGACUGUGAUGUG	22	186
sja-miR-novel-04	UAUCACAGUCCUGCUUAGGUGA	22	51
sja-miR-novel-05-5p	GCAGGAGUUUGAUUUCACAU	20	4
sja-miR-novel-05-3p	AAACAGACAUACCAAUGCAG	20	4
sja-miR-novel-06-5p	UUCUUUACAGUGGGUCUCUUU	21	4
sja-miR-novel-06-3p	GGUGAUCUUUGUAUGGACAA	20	4
sja-miR-novel-07	AUGCGCACUGCCGAGGAUUUC	21	6
sja-miR-novel-08	AUGCCUGACCACCGUCUACUU	21	5
sja-miR-novel-09-5p	UUAGGUUUCGUUGUUUGUAUUU	22	3
sja-miR-novel-09-3p	AUACAAACAGCUAAACUUAU	20	3
sja-miR-novel-10	GUUUACUGACGAAAGGACGCAU	22	4
sja-miR-novel-11-5p	UCUCUGUGUUGAAUUUGA	18	2
sja-miR-novel-11-3p	AGGUCUAUUGAUGCUGAGAUU	21	3
sja-miR-novel-12	UAUCACAGUCCAAGCUUUGGU	21	235

*In several cases, mature miRNAs from both 5′ and 3′ arms of the hairpin precursor are frequently identified. Such miRNAs are given names of the form sja-miR-novel-05-5p and sja-miR-novel-05-3p, and both are retained in the miRNA set in the study.

### Experimental validation of novel miRNAs

**Figure 2:**
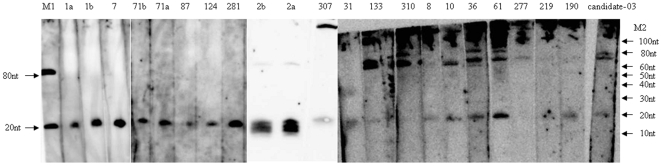
Northern analysis of conserved miRNAs expression. RNA (10 µg) isolated from *S. japonicum* schistosomula were separated on a 15% denaturing PAGE gel and transferred to a nylon membrane. Membranes were incubated with biotin-labeled probes complementary to candidate miRNA sequences (sja-miR-1a, sja-miR-1b, sja-miR-7, sja-miR-71b, sja-miR-71a, sja-miR-87, sja-miR-124, sja-miR-281, sja-miR-2b, sja-miR-2a, sja-miR-307, sja-miR-31, sja-miR-133, sja-miR-310, sja-miR-8, sja-miR-10, sja-miR-36, sja-miR-61, sja-miR-277, sja-miR-219, sja-miR-190, sja-miR-candidate-03). The detection of hybridization at 20 nucleotides indicates the expression of the corresponding sequences. Northern analysis with several of the probes do not reveal hybridization at 20nt indicating either that they are expressed at too low a level to be detected or that they are not expressed at all. M1: biotin-labeled marker; M2: 10bp DNA marker.

All *S. japonicum* miRNAs were tested by northern blotting to verify their expression. Eighteen conserved miRNAs and one candidate schistosome-specific miRNA gave a hybridization signal of approximately 22 nt (Figure 2). In some cases (such as sja-miR-10, sja-miR-36, sja-miR-61, sja-miR-133, sja-miR-277, sja-miR-310, and sja-miR-candidate-03), a presumed precursor transcript of about 80 nt was detected by northern blot in addition to the 22 nt species. A presumed precursor transcript of sja-miR-307 had a signal at 120 nt. The remaining 2 conserved and 16 schistosome-specific miRNAs could not be detected using northern blot analysis. We verified the expression of these miRNAs using stem-loop qRT-PCR (Figure S1).

### Read count as a relative measure of miRNAs abundance

The read count generated by deep sequencing is sometime used as a measure of relative miRNA expression levels. As shown in Table 2, different miRNAs were detected at different frequencies ranging from 2 (miR-133) to 584,105 (miR-1a) read counts. Moreover, the relative abundance of different members of the same miRNA family also varied greatly. For instance, miR-1a and miR-1b had 584,105 and 552 read counts, respectively, while miR-2a and miR-2b had only 13,431 and 57 read counts, respectively. It has been suggested that the dissimilarity of expression profiling for the miR1 and 2 families may be due to the pre-miRNA loop controlling or the result of the different functional roles of mature miRNAs [37].

The obvious differences in read counts described above were not always consistent with the signals derived by northern blot analysis. As shown in Figure 2 and Table 2, the read counts of sja-miR-1a and sja-miR-307 were 584,105 and 4 , respectively, whereas they show almost the same signal by northern blotting. It is possible that neither read counts nor northern blot analysis accurately reflect the relative abundance and expression levels of the miRNAs in vivo. The same problems were recently observed in both *Arabidopsis thaliana* [38] and porcine miRNAs [39]. Stem-loop qRT-PCR was also used to verify the expression of novel miRNAs [16],[27]. We were unable to support the idea of a correlation between the frequency of read counts and relative abundance in the RNA population by a complementary method.

### *S. japonicum* miRNA gene clusters

Studies of the transcription of miRNAs have shown that when several miRNAs genes are located in close proximity in the genome (i.e. clustered) they are often transcribed as a unit yielding a single polycistronic transcript [33]. It has been suggested that the different miRNAs are transcribed together because they are related to a single biological phenomenon. The extent of gene clustering is therefore considered to be of descriptive value that is suggestive of a relationship among the individual genes. There is, however, no universal definition of how close genes need to be in order to consider them a cluster.

We investigated the genomic arrangement of the miRNA genes identified in our study in an attempt to identify S. *japonicum* miRNA gene clusters. We used same criteria described previously [40],[41] in an attempt to identify the best candidates for subsequent studies on transcription. MiRNA genes located within 500 bp were assumed to be good candidates for further transcriptional studies.

**Figure 3:**
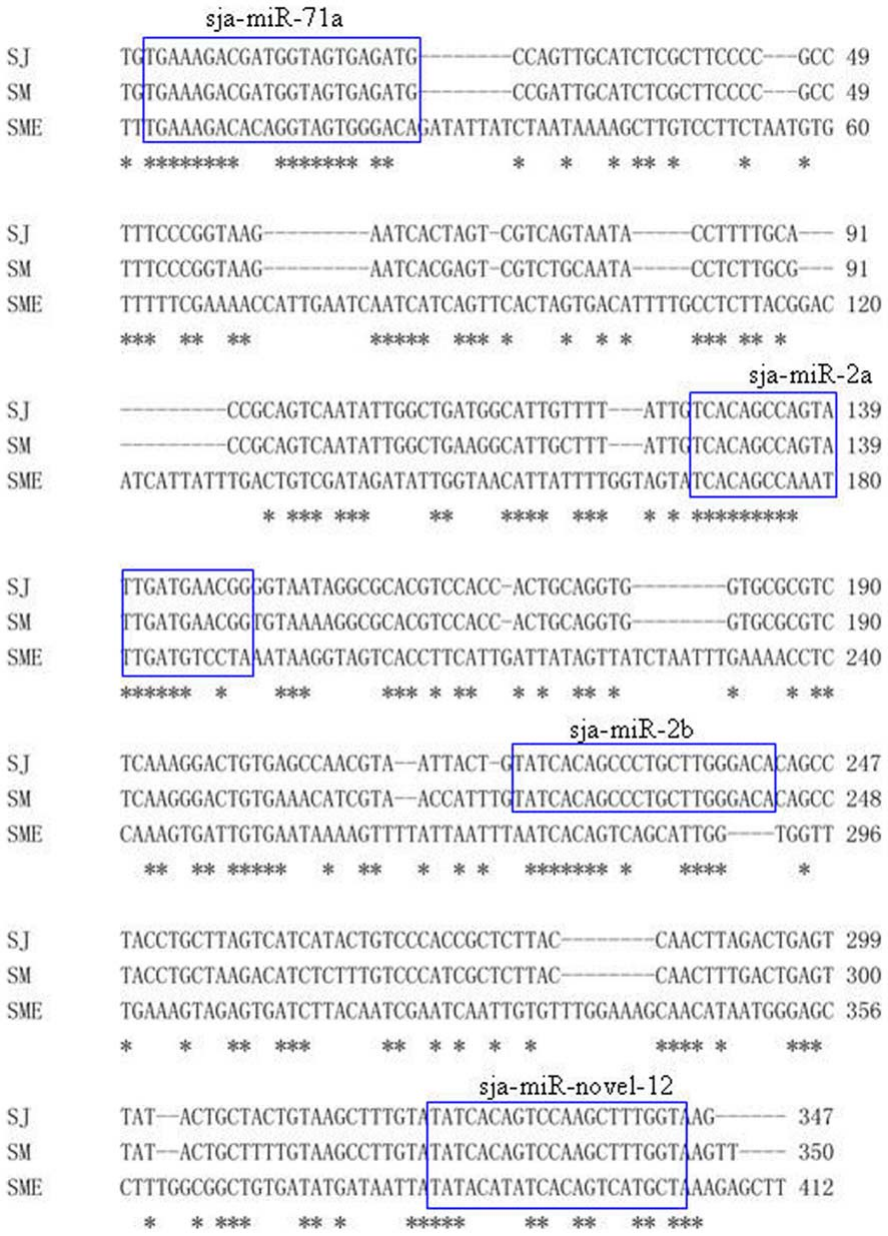
Conservation of the clustered arrangement miRNA genes among species. An alignment of genomic sequences from *S. japonicum* (EMBL:CABF01007682); *S. mansoni* (EMBL:FN357443); *S. mediterranea* (Smed_assembly_v31.001374, Accession numbers of the genome(contig) database of *S. mediterranea* from http://smedgd.neuro.utah.edu/) indicate that three conserved miRNA genes(sja-miR-71a, sja-miR-2a, sja-miR-2b) and one miRNA gene(sja-miR-novel-12), first reported here, are clustered together within approximately 350 base pair of each other. Accession numbers are given in parentheses. The sequences of mature miRNAs are boxed. The asterix denotes a nucleotide position that is conserved among species.

Based on the above criterion, 7 miRNAs were tentatively assigned to two clusters: miR-71a and miR-71b with 347 and 420 bp sequence ranges, respectively. Cluster miR-71a contains 4 stem-loop structures encoding miR-71a, miR-2a, miR-2b, and a schistosome-specific miRNA (sja-miR-novel-12) that shares the seed sequence of the miR-13 family which has been extensively studied in other organisms. Cluster miR-71b contains three miRNA members, the miR-71b, and two schistosome-specific miRNAs (sja-miR-novel-03 and sja-miR-novel-04). Interestingly, the nucleotide sequences and organization of cluster miR-71a contains four miRNAs which are likewise clustered within the *S. japonicum*, *S. mansoni*, and *S. mediterranea* genomes (Figure 3), The conserved association of the four genes in cluster miR-71a may indicate that they have interrelated functional roles in these organisms [40].

We found no direct evidence of polycistronic transcripts that encode the clustered genes described above. We therefore looked for other features of transcription that would associate the members of a cluster. One might expect that if a polycistronic transcript did exist which carried multiple members of a miRNA family the mature RNAs would be present in equal abundance in the existing RNA population although there are many exceptions to the rule. Relationships among different miRNAs assigned to a cluster were therefore investigated by determining the relative expression levels of the clustered miRNAs by qRT-PCR. The expression of sja-miR-2a and sja-miR-2b were found to be 31% and 26% of sja-miR-71a. This degree of variation in expression levels of miRNAs found in the same cluster has previously been reported [42],[43].

### Hairpin characteristics of *S. japonicum* miRNA

The length of miRNA precursor fold-back in animals is most often in the range of 60–80 nt, whereas in plants the lengths are more variable and may include up to a few hundred nt [44],[45]. In this study, we found that the *S. japonicum* miRNA precursor identified by homology searches would be predicted to form a structure consistent with miRNA precursors of >100 nt (Table S3), and a predicted hairpin of 60–100 nt.

For example, miR-307 sequence with approximately 60–80 nt of flanking sequence cannot form a typical hairpin conformation.

When the flanking sequence was expanded to 150 nt, a relatively stable stem-loop pre-miRNA was predicted (Δ*G*°_folding_ = −42.10 kcal/mol) by the RNA fold algorithm. Furthermore, northern blot analysis (Figure 2) demonstrated that there was a hybridization signal for miR-307 in the size range of 120 nucleotides in addition to the 22 nt signal resulting from hybridization to the mature miRNA. In general, this observation is consistent with the idea that the length of metazoan pre-miRNA sequences can be much longer than previously recognized [31].

### Identification of endogenous siRNAs

The existence of candidate endogenous siRNAs can also be based on the structural characteristics of this class of small RNAs. The endo-siRNAs have been extensively investigated in *C. elegans, Drosophila melanogaster* and mice [24], [46]–[53] and a number of properties appear to be consistent. Most of these endo-siRNA appear to be derived from transposable elements, complementary annealed transcripts, and long ‘fold-back’ transcripts called hairpin RNAs (hpRNAs) [47]. Small RNAs from our library were therefore screened to find candidate schistosome endo-siRNAs.

As shown in Table S6, 59669 reads (4858 unique sequences) were found which were fully complementary to mRNAs over a 20 nt region. The size distribution of these endo-siRNA candidates averaged between 20 and 21 nt, slightly less than the 22 nt average lengths of miRNAs and the 27 nt average lengths of piRNAs (Table S6). This observation is consistent with other reports [54]. We also found that about 40% of classes of the endo-siRNA candidates (30/76) are related to retrotransposon and retrovirus Pol polyprotein from transposon which is consistent with that proposal that these molecules are schistosome endo-siRNAs.

**Figure 4:**
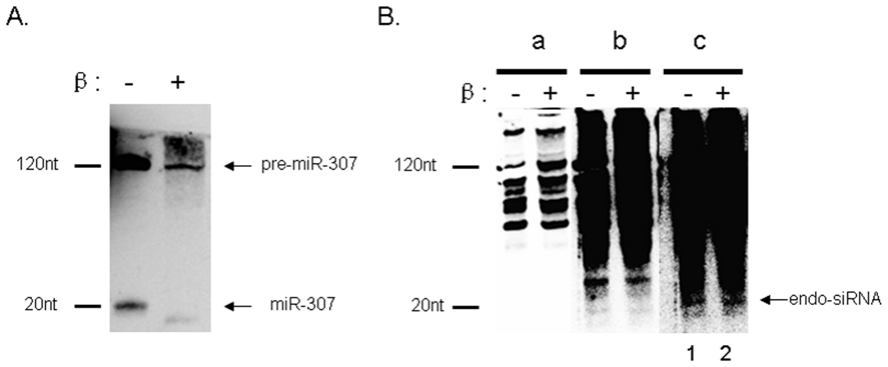
Sensitivity of the 3′ termini of small RNAs to periodate oxidation. RNAs terminating with a ribose are subject to periodate oxidation and subsequent β­elimination when both hydroxyl groups are unmodified and in a cis configuration. The reaction results in the loss of a single nucleoside at the 3′ position and changes its charge/mass ratio. Modification of the 3′ termini (e.g. 2′ O-methyl groups), prevents a RNA molecule from losing its terminal nucleoside. (**A**) Northern blot analysis of total RNAs of schistosomulum probed for miR-307 before (−) and after (+) periodate oxidation and β-elimination. (**B**) Northern blot analysis of total RNAs of schistosomulum probed for E3 (Endo-siRNA-3). Three different exposures of the same blot are shown( a, b and c) which show an increasingly complex pattern of bands as might be expected for transposon related sequence. Lane 1 represents untreated RNA in each exposure. Lane 2 represents RNA that has been treated by periodate oxidation and β-elimination. Mature siRNA hybrization is not seen in panel (a) and detection of a small shift in mobility in the larger RNA would not be expected. Panel (b) indicates the presence of smaller RNAs whose mobility is not affected by periodate treatment are seen but they are too large to be mature endo-siRNAs, Panel (c) indicates the presence of a 20 nt RNA that is not sensitive to periodate treatment. This supports the proposal that it is an endo-siRNA.

A characteristic that distinguishes both siRNAs and piRNAs from other small RNAs is the presence of a 2′-O-methyl modification on the 3′ terminal nucleotide [24],[51]. This difference can serve as the basis for distinguishing different small RNA molecules. The presence of a 2′-O-methyl modifications changes the sensitivity of RNA to periodate oxidation that only occurs when the cis-diol of the terminal ribose is unmodified. Oxidized RNAs lose their terminal residue under basic conditions as a result of β-elimination while RNA that is resistant to oxidation remains unchanged in size. The process thereby changes the charge/mass ratio of the RNAs that were unmodified and in so doing alter their electrophoretic mobility. Thus endo-siRNAs will be resistant to periodate oxidation and β-elimination while a miRNA will not. Treatment of schistosome endo-siRNA candidates in this manner indicated that they were modified at their 3′termini while the miRNAs were not (Figure 4 A and B). We have not determined the identity of the large number of higher molecular weight bands (in the lanes 1,2,) (Figure 4B). Given their size, resistance to periodate oxidation and sequence similarity with transposon and retroviral associated proteins we can tentatively assign a number of small RNAs to the endo-siRNAs class of molecules.

## Discussion

Messenger RNAs were once thought to contain the majority of the informational content of the genome. We were able to use limited amounts of sequence data to gain a basic understanding of the processes of transcription and translation. For example, restrictions on genetic drift imposed by the genetic code permitted us not only to define genes, protein structure and functional motifs but also permitted us to gain a greater understanding of transcriptional factors and protein structure. The discovery that families of noncoding RNA species played a critical role in many biological processes presented new challenges for biologists in that primary sequence did not yield as much easily interpretable information. For example, determination of the function of a noncoding RNA in one organism often did not provide the information necessary to identify its counterpart in another. The challenge then became to describe consistent features of the different types of non-coding RNA that would serve to identify them. Dramatic advances in sequencing technology have not only provided the data to approach this challenge but also have opened up new dimensions in the study of biological control by allowing us to rapidly characterize RNA population in depth as opposed to simply defining a few representative individuals from the population. The dramatic increase in the number of individual molecules that can now be sampled from a single population has enabled us to detect changes in the relative frequencies of individual RNAs, to describe the course precursor processing pathways and to identify changes in RNA structure due to post transcriptional processes.

The study of *S. japonicum* small RNA presented here displays the potential of high-throughput sequencing to shed light on the biological control mechanisms of an important pathogen. Recently 5 miRNAs were identified in adult worms by traditional cloning approaches in our laboratory [16] and several candidate *S. mansoni* miRNAs were reported using computational approaches [35] While the current article was in preparation, Copeland et al. [55]reported the discovery of two additional miRNAs in *S. mansoni* on the basis of an homology search. One of these is also conserved in *S. japonicum* while the other one is not. In this study, we systematically investigated miRNAs from the schistosomulum stage of the *S. japonicum* life cycle and the possibility of endo-siRNA involvement in the biological control of the organism. Using deep sequencing and computational analysis, we identified 36 novel conserved and *Schistosoma*-specific miRNAs and described two clustered groups of miRNAs genes in the genome. We also found a family of small RNA that have the characteristics of endogenously produced siRNAs( endo-siRNAs) and appear to target retrotransposons and retrovirus-related Pol polyprotein from transposon.

About 56% of *S. japonicum* miRNAs were categorized as conserved(i.e. sequence identify to *S. mediterranea* and *D. melanogaster*) and accounted for 90% of the read counts, whereas the remaining *S.japonicum* miRNAs were categorized as *Schistosoma*-specific miRNAs and accounted for <10% of the read counts. This observation is consistent with current dogma suggesting that species-specific miRNAs are expressed at a lower level than conserved miRNAs. It is plausible that the conserved miRNAs are responsible for control of the basic developmental pathways in most eukaryotes, while nonconserved miRNAs are involved in regulation of species-specific pathways and functions [18].

The number of confidently identified miRNA genes has reached 110 in *C. elegans* and 71 in *S. mediterranea*, accounting for about 1–2% of the predicted genes in the genome [24],[40]. If miRNA's represent a similar percentage of *S. japonicum* genes, the investigation of small RNAs from other forms of the parasite (such as egg and cercaria) should reveal both new conserved and *Schistosoma*-specific miRNAs in concert with the organisms life cycle. We have also made substantial progress towards understanding the pathway involved in the production of mature miRNA from *S. japonicum* including defining precursor structures and RNA fragments resulting from the maturation process. Given the process of miRNA production varies to a significant degree among species, advances in these areas will be essential to the development of antisense oligonucleotide interference(ASO) technology in this organism.

In addition to identifying specific miRNAs as defined by a single primary sequence(the reference sequence), we have observed different degrees of heterogeneity at both 3′ and 5′ ends of these molecules. Such variability may be the result of imprecise or alternative processing by Drosha or Dicer although PCR amplification error can not be ruled out [24]. The biological impact of miRNA heterogeneity is still a matter of question but it has also been observed in other studies [17],[24],[31]. We do know that changes in the termini can dramatically affect which strand of the RNA duplex produced by Dicer activity is selected to be the miRNA and thus the identity of the seed sequence [31].

The identification of miRNA clusters which include orthologs whose function has been identified in model organism may provide insight into their function in *S. japonicum* [56]. For example, the miR-71a cluster of *S. japonicum* contains miRNA sequences that have been associated with the suppression of apoptosis [57]–[59] in both Drosophila and silkworms. It also merit mention that the genomic arrangement of miRNAs, including miR-71a, the miR-2 family and sja-miR-novel-12 (miR-13), is conserved in *S. mediterranea* and *S. mansoni*. This preservation of the clustered arrangement in the three organisms may indicate restraints on their genomic organization.

Introducing exogenous siRNA directly or as part of a plasmid is often a useful approach to defining protein function. In theory, any gene of known sequence could be targeted by an appropriately designed siRNA construct. The problems associated with exclusive targeting of a single mRNA on the basis of sequence complementarity are many and until recently it was thought that these molecules were rarely employed as a natural means of control. Recently, however, endogenously produced siRNAs have been found in *C. elegans*, *Drosophila* and mice [24],[47],[53]. These observations give hope that siRNA can be used theraputically but much will depend upon studying how they are employed for biological control in organisms where they are endogenously produced.

In this study, we report for the first time the presence of endo-siRNAs in the schistosome species *S. japonicum*. These endo-siRNAs candidates were selected on the basis of scanning sequences reported here with all annotated *S. japonicum* mRNAs genes. A large number were found which averaged 22 nt in length.and were fully complementary to *S. japonicum* exons. Forty percent of the identified RNA sequences were complementary to a small subset of retrotransposon and retrovirus-related Pol polyprotein related sequence. Further these small RNAs have a modified 3′ terminus that is characteristic of the 2′-O-methyl modifications of endo-siRNAs. These small RNAs are therefore related as a family and have all the characteristics associated with the endo-siRNA group described in other organisms.

A RNA population 20 to 120 nucleotides in length was detected by northern blot analysis when the putative schistosome endo-siRNAs sequences were used as a probe(Figure 4). This is reminiscent of data from studies describing promoter-associated RNAs [60]–[62]. Although we have not determined the identity of the large number of higher molecular weight bands (Figure 4B in the lanes 1,2), it seems likely that schistosome endo-siRNAs are derived from a precursor with bidirectional convergent and divergent transcription [46],[63]. Core et al. proposed that transcription start site-associated RNAs (TSSa-RNAs) that result from divergent transcription could themselves be functional *via* either Argonaute-dependent or -independent pathways [60].

In summary, deep sequencing has provided useful information regarding the small RNAs population in *S. japonicum*, an important worldwide pathogen. This study led to the discovery of 16 schistosome-specific miRNA families in addition to 20 conserved miRNA families that have homologs in other organisms. We further show two miRNA clusters that are highly conserved among *S. japonicum*, *S. mansoni*, and *S. Mediterranea*. Moreover, we identified a family of small RNAs that appear to be endo-siRNAs associated with retrotransposon and retrovirus-related Pol polyprotein from transposons. Our study should serve as a foundation for future studies aimed at understanding the functions of small RNAs and their role in the regulatory networks

## Supporting Information

Figure S1 Amplification plots of 16 schistosome-specific miRNAs. MiRNAs from left to right are as follows: A: sja-miR-novel-03,sja-miR-novel-10,sja-miR-novel-11-5p; B: sja-miR-novel-01, sja-miR-novel-04, sja-miR-novel-11-3p; C: sja-miR-novel-12, sja-miR-novel-07, sja-miR-novel-02; D: sja-miR-novel-05-5p, sja-miR-novel-08, sja-miR-novel-05-3p; E: sja-miR-novel-09-5p, sja-miR-novel-06-5p, sja-miR-novel-06-3p; F: sja-miR-novel-09-3p, sja-miR-1a. The same amount of cDNA was added to each qRT-PCR reaction. Amplification of sja-miR-1a was used as a positive control.
(3.53 MB TIF)

Table S1 Statistic analysis of sRNA sequences against the *S. japonicum* and *S. mansoni* reference genomes.
(0.02 MB XLS)

Table S2 Predicted secondary structure of IsomiRs for five known miRNAs.
(0.03 MB XLS)

Table S3 Predicted secondary structure of IsomiRs for 20 conserved miRNAs.
(0.12 MB XLS)

Table S4 Predicted secondary structure of IsomiRs for 16 schistosome-specific miRNAs.
(0.04 MB XLS)

Table S5 Predicted secondary structure of IsomiRs for 21 miRNA candidates.
(0.05 MB XLS)

Table S6 Sequences of schistosome endo-siRNAs and predicted functions.
(0.57 MB XLS)

Table S7 Sequences of the probe used for northern analysis.
(0.02 MB XLS)

Table S8 Sequences of the primers used for stem-loop RT-PCR.
(0.03 MB XLS)

## References

[1] Ivey KN, Muth A, Arnold J, King FW, Yeh RF (2008). MicroRNA regulation of cell lineages in mouse and human embryonic stem cells.. Cell Stem Cell.

[2] Kim VN (2005). Small RNAs: classification, biogenesis, and function.. Mol Cells.

[3] Malone CD, Hannon GJ (2009). Small RNAs as guardians of the genome.. Cell.

[4] Ghildiyal M, Zamore PD (2009). Small silencing RNAs: an expanding universe.. Nat Rev Genet.

[5] Denli AM, Tops BB, Plasterk RH, Ketting RF, Hannon GJ (2004). Processing of primary microRNAs by the Microprocessor complex.. Nature.

[6] Lee Y, Ahn C, Han J, Choi H, Kim J (2003). The nuclear RNase III Drosha initiates microRNA processing.. Nature.

[7] Ketting RF, Fischer SEJ, Bernstein E, Sijen T, Hannon GJ (2001). Dicer functions in RNA interference and in synthesis of small RNA involved in developmental timing in C-elegans.. Genes & Development.

[8] Schwarz DS, Hutvagner G, Du T, Xu ZS, Aronin N (2003). Asymmetry in the assembly of the RNAi enzyme complex.. Cell.

[9] Flynt AS, Lai EC (2008). Biological principles of microRNA-mediated regulation: shared themes amid diversity.. Nature Reviews Genetics.

[10] Tomari Y, Zamore PD (2005). Perspective: machines for RNAi.. Genes & Development.

[11] Farazi TA, Juranek SA, Tuschl T (2008). The growing catalog of small RNAs and their association with distinct Argonaute/Piwi family members.. Development.

[12] Kim VN, Han J, Siomi MC (2009). Biogenesis of small RNAs in animals.. Nature Reviews Molecular Cell Biology.

[13] Vagin VV, Sigova A, Li CJ, Seitz H, Gvozdev V (2006). A distinct small RNA pathway silences selfish genetic elements in the germline.. Science.

[14] Zhou Y, Zheng HJ, Chen YY, Zhang L, Wang K (2009). The Schistosoma japonicum genome reveals features of host-parasite interplay.. Nature.

[15] Berriman M, Haas BJ, LoVerde PT, Wilson RA, Dillon GP (2009). The genome of the blood fluke Schistosoma mansoni.. Nature.

[16] Xue X, Sun J, Zhang Q, Wang Z, Huang Y (2008). Identification and characterization of novel microRNAs from Schistosoma japonicum.. PLoS ONE.

[17] Morin RD, O'Connor MD, Griffith M, Kuchenbauer F, Delaney A (2008). Application of massively parallel sequencing to microRNA profiling and discovery in human embryonic stem cells.. Genome Research.

[18] Glazov EA, Cottee PA, Barris WC, Moore RJ, Dalrymple BP (2008). A microRNA catalog of the developing chicken embryo identified by a deep sequencing approach.. Genome Research.

[19] Lu C, Meyers BC, Green PJ (2007). Construction of small RNA cDNA libraries for deep sequencing.. Methods.

[20] Lopez R, Silventoinen V, Robinson S, Kibria A, Gish W (2003). WU-Blast2 server at the European Bioinformatics Institute.. Nucleic Acids Res.

[21] Gardner PP, Daub J, Tate JG, Nawrocki EP, Kolbe DL (2009). Rfam: updates to the RNA families database.. Nucleic Acids Research.

[22] Hofacker IL (2003). Vienna RNA secondary structure server.. Nucleic Acids Research.

[23] Gruber AR, Lorenz R, Bernhart SH, Neuboock R, Hofacker IL (2008). The Vienna RNA Websuite.. Nucleic Acids Research.

[24] Ruby JG, Jan C, Player C, Axtell MJ, Lee W (2006). Large-scale sequencing reveals 21U-RNAs and additional microRNAs and endogenous siRNAs in C-elegans.. Cell.

[25] Ambros V, Lee RC, Lavanway A, Williams PT, Jewell D (2003). MicroRNAs and other tiny endogenous RNAs in C-elegans.. Current Biology.

[26] Lau NC, Lim LP, Weinstein EG, Bartel DP (2001). An abundant class of tiny RNAs with probable regulatory roles in Caenorhabditis elegans.. Science.

[27] Chen CF, Ridzon DA, Broomer AJ, Zhou ZH, Lee DH (2005). Real-time quantification of microRNAs by stem-loop RT-PCR.. Nucleic Acids Research.

[28] Livak KJ, Schmittgen TD (2001). Analysis of relative gene expression data using real-time quantitative PCR and the 2(T)(-Delta Delta C) method.. Methods.

[29] Horwich MD, Li CJ, Matranga C, Vagin V, Farley G (2007). The Drosophila RNA methyltransferase, DmHen1, modifies germline piRNAs and single-stranded siRNAs in RISC.. Current Biology.

[30] Alefelder S, Patel BK, Eckstein F (1998). Incorporation of terminal phosphorothioates into oligonucleotides.. Nucleic Acids Research.

[31] Ruby JG, Stark A, Johnston WK, Kellis M, Bartel DP (2007). Evolution, biogenesis, expression, and target predictions of a substantially expanded set of Drosophila microRNAs.. Genome Research.

[32] miRBase.[http://microrna.sanger.ac.uk/registry/]

[33] Griffiths-Jones S, Saini HK, van Dongen S, Enright AJ (2008). miRBase: tools for microRNA genomics.. Nucleic Acids Research.

[34] Yao YY, Guo GG, Ni ZF, Sunkar R, Du JK (2007). Cloning and characterization of microRNAs from wheat (Triticum aestivum L.).. Genome Biology.

[35] Hertel J, Lindemeyer M, Missal K, Fried C, Tanzer A (2006). The expansion of the metazoan microRNA repertoire.. Bmc Genomics.

[36] Bar M, Wyman SK, Fritz BR, Qi JL, Garg KS (2008). MicroRNA Discovery and Profiling in Human Embryonic Stem Cells by Deep Sequencing of Small RNA Libraries.. Stem Cells.

[37] Liu G, Min H, Yue S, Chen CZ (2008). Pre-miRNA loop nucleotides control the distinct activities of mir-181a-1 and mir-181c in early T cell development.. PLoS ONE.

[38] Rajagopalan R, Vaucheret H, Trejo J, Bartel DP (2006). A diverse and evolutionarily fluid set of microRNAs in Arabidopsis thaliana.. Genes & Development.

[39] Reddy AM, Zheng Y, Jagadeeswaran G, Macmil SL, Graham WB (2009). Cloning, characterization and expression analysis of porcine microRNAs.. BMC Genomics.

[40] Palakodeti D, Smielewska M, Graveley BR (2006). MicroRNAs from the Planarian Schmidtea mediterranea: A model system for stem cell biology.. Rna-a Publication of the Rna Society.

[41] Lu C, Tej SS, Luo SJ, Haudenschild CD, Meyers BC (2005). Elucidation of the small RNA component of the transcriptome.. Science.

[42] Barad O, Meiri E, Avniel A, Aharonov R, Barzilai A (2004). MicroRNA expression detected by oligonucleotide microarrays: system establishment and expression profiling in human tissues.. Genome Res.

[43] Sempere LF, Freemantle S, Pitha-Rowe I, Moss E, Dmitrovsky E (2004). Expression profiling of mammalian microRNAs uncovers a subset of brain-expressed microRNAs with possible roles in murine and human neuronal differentiation.. Genome Biol.

[44] Bartel DP (2004). MicroRNAs: Genomics, biogenesis, mechanism, and function.. Cell.

[45] Ambros V, Bartel B, Bartel DP, Burge CB, Carrington JC (2003). A uniform system for microRNA annotation.. Rna.

[46] Okamura K, Balla S, Martin R, Liu N, Lai EC (2008). Two distinct mechanisms generate endogenous siRNAs from bidirectional transcription in Drosophila melanogaster.. Nat Struct Mol Biol.

[47] Okamura K, Chung WJ, Ruby JG, Guo HL, Bartel DP (2008). The Drosophila hairpin RNA pathway generates endogenous short interfering RNAs.. Nature.

[48] Chung WJ, Okamura K, Martin R, Lai EC (2008). Endogenous RNA interference provides a somatic defense against Drosophila transposons.. Curr Biol.

[49] Czech B, Malone CD, Zhou R, Stark A, Schlingeheyde C (2008). An endogenous small interfering RNA pathway in Drosophila.. Nature.

[50] Ghildiyal M, Seitz H, Horwich MD, Li C, Du T (2008). Endogenous siRNAs derived from transposons and mRNAs in Drosophila somatic cells.. Science.

[51] Kawamura Y, Saito K, Kin T, Ono Y, Asai K (2008). Drosophila endogenous small RNAs bind to Argonaute 2 in somatic cells.. Nature.

[52] Tam OH, Aravin AA, Stein P, Girard A, Murchison EP (2008). Pseudogene-derived small interfering RNAs regulate gene expression in mouse oocytes.. Nature.

[53] Watanabe T, Totoki Y, Toyoda A, Kaneda M, Kuramochi-Miyagawa S (2008). Endogenous siRNAs from naturally formed dsRNAs regulate transcripts in mouse oocytes.. Nature.

[54] Golden DE, Gerbasi VR, Sontheimer EJ (2008). An inside job for siRNAs.. Molecular Cell.

[55] Copeland CC, Marz M, Rose D, Hertel J, Brindley PJ (2009). Homology-based annotation of non-coding RNAs in the genomes of Schistosoma mansoni and Schistosoma japonicum.. BMC Genomics.

[56] Ambros V (2004). The functions of animal microRNAs.. Nature.

[57] Leaman D, Chen PY, Fak J, Yalcin A, Pearce M (2005). Antisense-mediated depletion reveals essential and specific functions of microRNAs in Drosophila development.. Cell.

[58] Enright AJ, John B, Gaul U, Tuschl T, Sander C (2003). MicroRNA targets in Drosophila.. Genome Biol.

[59] Yu X, Zhou Q, Li SC, Luo Q, Cai Y (2008). The silkworm (Bombyx mori) microRNAs and their expressions in multiple developmental stages.. PLoS ONE.

[60] Core LJ, Waterfall JJ, Lis JT (2008). Nascent RNA Sequencing Reveals Widespread Pausing and Divergent Initiation at Human Promoters.. Science.

[61] Seila AC, Calabrese JM, Levine SS, Yeo GW, Rahl PB (2008). Divergent Transcription from Active Promoters.. Science.

[62] Buratowski S (2008). Transcription. Gene expression–where to start?. Science.

[63] Okamura K, Lai EC (2008). Endogenous small interfering RNAs in animals.. Nature Reviews Molecular Cell Biology.

