# Supplementary figures and images for: An “In-Depth” Description of the Small Non-coding RNA Population of Schistosoma japonicum Schistosomulum
Source: PLoS Negl Trop Dis. 2010 Feb 9;4(2):e596. doi: 10.1371/journal.pntd.0000596 (PMC2817716; doi:10.1371/journal.pntd.0000596)

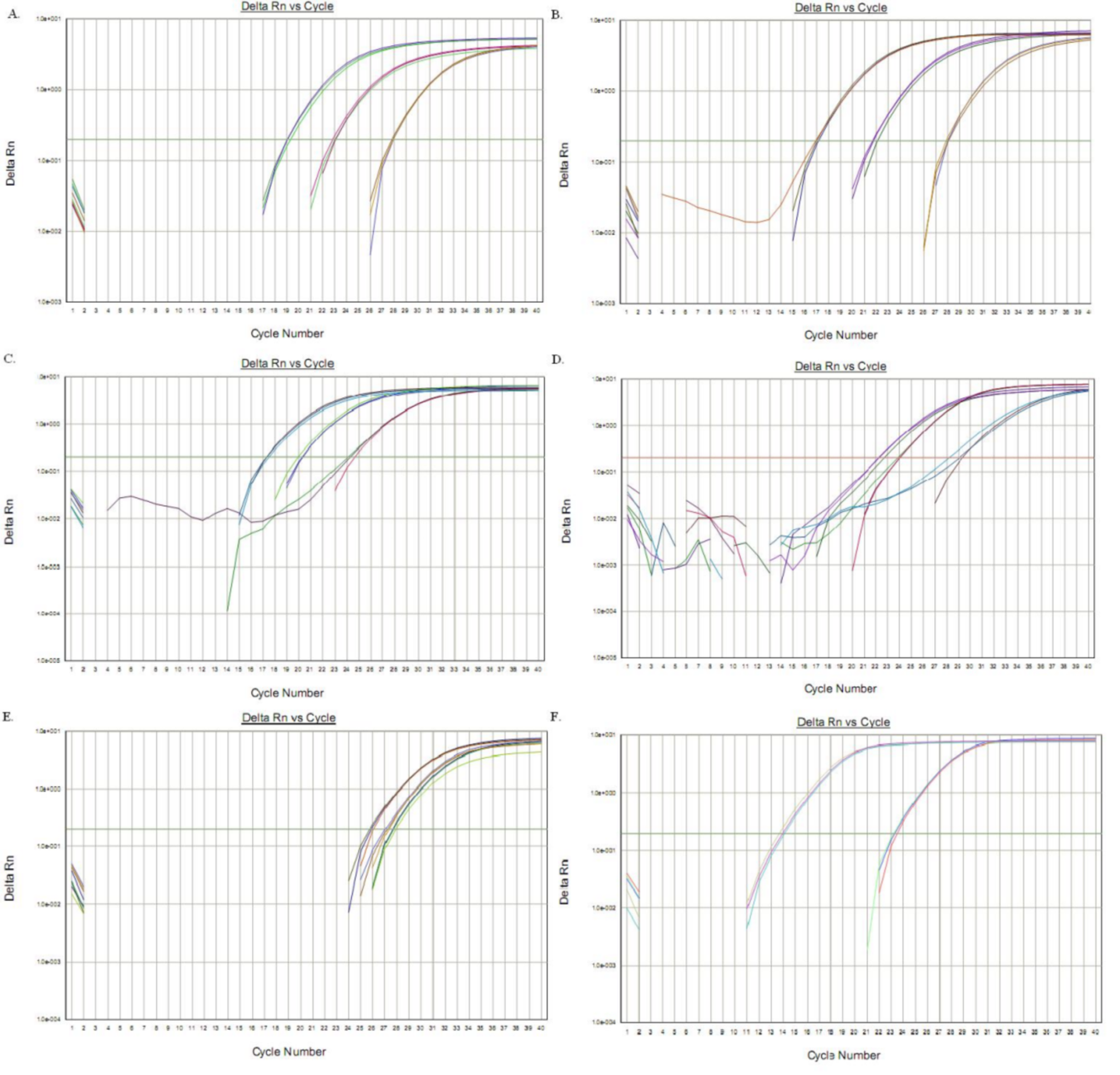

Supplement: Figure S1 — Amplification plots of 16 schistosome-specific miRNAs. MiRNAs from left to right are as follows: A: sja-miR-novel-03,sja-miR-novel-10,sja-miR-novel-11-5p; B: sja-miR-novel-01, sja-miR-novel-04, sja-miR-novel-11-3p; C: sja-miR-novel-12, sja-miR-novel-07, sja-miR-novel-02; D: sja-miR-novel-05-5p, sja-miR-novel-08, sja-miR-novel-05-3p; E: sja-miR-novel-09-5p, sja-miR-novel-06-5p, sja-miR-novel-06-3p; F: sja-miR-novel-09-3p, sja-miR-1a. The same amount of cDNA was added to each qRT-PCR reaction. Amplification of sja-miR-1a was used as a positive control. (3.53 MB TIF) [file pntd.0000596.s001.tif]
